# The CK1δ/ϵ-Tip60 Axis Enhances Wnt/β-Catenin Signaling *via* Regulating β-Catenin Acetylation in Colon Cancer

**DOI:** 10.3389/fonc.2022.844477

**Published:** 2022-04-12

**Authors:** Jiong Ning, Qi Sun, Zijie Su, Lifeng Tan, Yun Tang, Sapna Sayed, Huan Li, Vivian Weiwen Xue, Shanshan Liu, Xianxiong Chen, Desheng Lu

**Affiliations:** ^1^ Guangdong Provincial Key Laboratory of Regional Immunity and Diseases, International Cancer Center, Department of Pharmacology, Shenzhen University Health Science Center, Shenzhen, China; ^2^ Shenzhen University-Friedrich Schiller Universität Jena Joint PhD Program in Biomedical Sciences, Shenzhen University School of Medicine, Shenzhen, China; ^3^ Department of Research, The Affiliated Tumor Hospital of Guangxi Medical University, Nanning, China

**Keywords:** CK1δ/ϵ, Tip60, β-catenin acetylation, Wnt/β-catenin signaling, colon cancer

## Abstract

Casein kinase 1δ/ϵ (CK1δ/ϵ) are well-established positive modulators of the Wnt/β-catenin signaling pathway. However, the molecular mechanisms involved in the regulation of β-catenin transcriptional activity by CK1δ/ϵ remain unclear. In this study, we found that CK1δ/ϵ could enhance β-catenin-mediated transcription through regulating β-catenin acetylation. CK1δ/ϵ interacted with Tip60 and facilitated the recruitment of Tip60 to β-catenin complex, resulting in increasing β-catenin acetylation at K49. Importantly, Tip60 significantly enhanced the SuperTopFlash reporter activity induced by CK1δ/ϵ or/and β-catenin. Furthermore, a CK1δ/CK1ϵ/β-catenin/Tip60 complex was detected in colon cancer cells. Simultaneous knockdown of CK1δ and CK1ϵ significantly attenuated the interaction between β-catenin and Tip60. Notably, inhibition of CK1δ/ϵ or Tip60, with shRNA or small molecular inhibitors downregulated the level of β-catenin acetylation at K49 in colon cancer cells. Finally, combined treatment with CK1 inhibitor SR3029 and Tip60 inhibitor MG149 had more potent inhibitory effect on β-catenin acetylation, the transcription of Wnt target genes and the viability and proliferation in colon cancer cells. Taken together, our results revealed that the transcriptional activity of β-catenin could be modulated by the CK1δ/ϵ-β-catenin-Tip60 axis, which may be a potential therapeutic target for colon cancer.

## Introduction

The Wnt/β-catenin signaling pathway plays crucial roles in embryonic development, tissue homeostasis, stem cell renewal and tumorigenesis (1, 2). In this pathway, β-catenin is a central component in Wnt signal transduction, acting as a transcriptional co-activator to mediate the expression of Wnt target genes (1). The stability and activity of β-catenin is regulated by post-translational modifications, such as phosphorylation, ubiquitination and acetylation (3). In the absence of Wnt stimulation, β-catenin exists in a complex, which is consisted of the adenomatosis polyposis coli protein (APC), the scaffolding protein axin, the casein kinase 1 (CK1) and the glycogen synthetase kinase-3β enzyme (GSK-3β). GSK-3β phosphorylates β-catenin and triggers its ubiquitination-mediated degradation. Upon stimulation with Wnt, GSK-3β dissociates from the destruction complex and unphosphorylated β-catenin accumulates in the cytosol and nucleus (4). As acetyltransferases, cAMP response element binding (CREB) binding protein (CBP), p300 and p300/CBP-associated factor (PCAF) could catalyze the acetylation of β-catenin at different residues (K19, K49 and K345), thereby promoting the stability of β-catenin, enhancing the interaction between β-catenin and TCF4, ultimately stimulating the transcription of target genes (5–8).

TAT-interactive protein 60 kDa (Tip60), a member of the Moz, Ybf2/Sas3, Sas2 and Tip60 (MYST) family of histone acetyltransferases (HATs), is a crucial regulator of the DNA damage response and transcriptional co-activator (9–11). This protein has been involved in a wide variety of cellular activities, including transcriptional regulation, DNA repair, checkpoint activation, apoptosis and autophagy (11, 12). Tip60 has been shown to acetylate histone and non-histone proteins, such as histones H2A, H3, H4, p53 and ataxia telangiectasia mutant (ATM) (13–15). Previous studies showed that reduced Tip60 expression was detected in colon, lung, breast, melanoma, prostate and gastric cancers (16–20). Interestingly, it was observed that lower levels of Tip60 may correlate with a worse prognosis. Tip60 has been found to drive prostate cancer proliferation by increasing the levels of c-Myc and androgen receptor (21). Stacy et al. reported that Tip60 could promote cellular proliferation by stabilizing ΔNp63α protein levels in squamous cell carcinoma (22). Moreover, several specific inhibitors of Tip60, including NU9056 and TH1834, have been developed and demonstrated to reduce proliferation of cancer cells (23, 24). However, the molecular mechanisms by which Tip60 influence cancer progression are not fully understood.

CK1 belongs to a family of serine/threonine protein kinases, composed of seven family members α, β, γ1, γ2, γ3, δ and ϵ in human. Among them, CK1δ and CK1ϵ share the highest homology, with 98% amino acid sequence identity in their kinase domain (25, 26). CK1 kinases are important regulators in diverse signaling pathways, including the Wnt signaling pathway (26). CK1 phosphorylates several key components in the Wnt signal cascade and exhibits both positive and negative roles (26). As a component of the β-catenin destruction complex, CK1α phosphorylates β-catenin at S45 and primes β-catenin for further phosphorylation of T41, S37, and S33 by GSK3β, resulting in the ubiquitin-proteasome-mediated degradation of β-catenin (26–28). Phosphorylation of low-density lipoprotein receptor-related protein 6 (LRP6) at T1479 and T1493 by CK1γ is required for the activation of Wnt/β-catenin signaling (29). CK1δ and CK1ϵ are well-known positive regulators of Wnt/β-catenin signaling (26, 30). In response to Wnt, CK1δ/ϵ binds to dishevelled (DVL) and phosphorylates DVL at multiple sites (31). CK1ϵ also phosphorylates LRP6 at T1493 and regulates initial steps in LRP6 signalosome formation (32, 33). A recent study showed that CK1δ/ϵ could regulate the stability of amino-terminal enhancer of split (AES), a member of the Groucho/transducin-like enhancer of split/Groucho-related gene (Gro/TLE/Grg) family (34). CK1δ/ϵ promoted S phase kinase-associated protein 2 (SKP2)-mediated ubiquitination and degradation of AES through phosphorylating AES at S121, resulting in the activation of Wnt/β-catenin signaling (34). However, very little is known about how CK1δ/ϵ is implicated in β-catenin-mediated transcriptional activity.

In this study, we explored the effect of CK1 on β-catenin-mediated Wnt signaling. We found that CK1δ/ϵ could increase β-catenin-mediated transcription through regulating β-catenin acetylation. We further investigated the molecular mechanism by which CK1δ/ϵ modulated the acetylation of β-catenin. Our results revealed that a novel CK1δ/ϵ-Tip60-β-catenin axis is involved in regulation of Wnt/β-catenin signaling.

## Methods and Materials

### Cell Culture

The human embryonic kidney 293T (HEK293T) cells, and colon cancer SW480 and HCT116 cells were obtained from American Type Culture Collection (ATCC, Manassas, VA, USA). These cells were maintained in DMEM (Thermo Fisher Scientific, Waltham, MA, USA) supplemented with 10% fetal bovine serum (FBS, Thermo Fisher Scientific, Waltham, MA, USA) and 1% penicillin-streptomycin (Thermo Fisher Scientific, Waltham, MA, USA) in a humidified incubator at 37°C with 5% CO_2_. Cells in the logarithmic phase of growth were used for the subsequent experiments.

### Chemical Regents, Antibodies and Plasmids

SR3029, D4476, MG149, NU9056, MG132 and cycloheximide (CHX) were purchased from MedChemExpress (MCE, Monmouth Junction, NJ, USA). The following primary antibodies were used: anti-CK1δ, anti-β-catenin, anti-p300, anti-acetyl-lysine, IgG (Santa Cruz Biotechnology, Heidelberg, Germany), anti-CBP, anti-acetyl-β-catenin (K49), anti-phospho-β-catenin (S45), anti-Flag, anti-V5, anti-CK1ϵ, anti-GFP, anti-mouse IgG (Cell Signaling Technology, Danvers, MA), anti-Tip60 (Abcam, Cambridge, MA, USA), and anti-GAPDH antibody (Proteintech, Chicago, IL, USA). The Tip60-Flag, PCAF-Flag, p300-Flag and pEGFP-N1 plasmids were purchased from Vigenebio (Weizhen, Shandong, China). The SuperTopFlash reporter plasmid was provided by Karl Willert (University of California at San Diego, La Jolla, CA, USA). The expression plasmids encoding β-catenin, pCMXβgal (β galactosidase, β-gal), CK1α-V5, CK1δ-V5, CK1ϵ-V5, CK1α-Flag, CK1δ-Flag, CK1ϵ-Flag, CK1γ-Flag have been described previously (35, 36). For the construction of GFP-tagged CK1δ, the cDNAs encoding human CK1δ was amplified by PCR and subcloned into the BamHI/EcoRI site of pEGFP-N1 vector using ClonExpress Ultra One Step Cloning Kit (Vazyme, Nanjing, China). The primer sequences used are as follows: CK1δ-GFP-sense, 5’-CTCGAGCTCAAGCTTCATGGAGCTGAGAGTCGGGAAC-3’; CK1δ-GFP-antisense, 5’-CTCACCATAAGGTGGCGACCGGTGTAGGTGCGTCGTG.

### Lentiviral Production and Infection

The CK1 shRNA oligos were cloned into pLKO.1-GFP vector, and the Tip60 shRNA oligos were inserted into pLKO.1-TRC vector. The resulting constructs were validated by sequencing. The shRNA oligos used have been previously described (37, 38). For lentiviral production and infection, HEK293T cells were transfected with a shRNA-expressing plasmid (10 µg), an envelope plasmid (pMD2.G, 2.5 µg) and a packaging plasmid (psPAX2, 7.5 µg) using Lipofectamine 2000 regent according to manufacturer’s instruction. At 48 h after transfection, viral supernatants were collected and filtered through a 0.45 μm filter. Virus was immediately added to SW480 and HCT116 cells with 8 µg/mL polybrene. After a week of infection, the GFP-positive cells were sorted by FACS AriaIII, and the knockdown of CK1δ or CK1ϵ was verified by Western blotting. For Tip60 deficient cells, cells were selected for stable expression in the presence of 3 µg/mL puromycin (Thermo Fisher Scientific, Waltham, MA, USA) after 72 h of infection. The puromycin-resistant stable clones were pooled, and Tip60 deficiency was confirmed by Western blotting.

### Luciferase Reporter Gene Assays

HEK293T cells were transfected with the SuperTopFlash reporter, control vector, and the indicated expression plasmids in 24-well plates using Lipofectamine 2000 (Thermo Fisher, San Jose, California, USA). After 48 h, the cells were harvested and lysates were used to examine the expression of luciferase by a luciferase assay kit (Promega, Shanghai, China), according to the manufacturer’s instructions. The luciferase values were normalized using the β-gal internal control to determine the variation in transfection efficiency. The results are presented as means ± SD of at least three independent experiments. The values for luciferase activity were presented as fold induction over control.

### Immunoblotting and Immunoprecipitation

The cells were lysed in RIPA buffer containing 50 mM Tris-HCl at pH 7.4, 150 mM NaCl, 1% Nonidet P-40, 0.1% SDS, 0.5% sodium deoxycholate, 1 mM EDTA, 1 mM PMSF, protease inhibitors (Bimake, Beijing, China), and phosphatase inhibitors (Topscience, Shanghai, China). After quantifying the concentration of protein using the BCA protein assay kit (Vazyme, Nanjing, China), 30 µg of protein was loaded and separated by SDS-PAGE, followed by transferring to poly-vinylidenefluoride (PVDF) membranes (Millipore, Massachusetts, USA). Immunoblotting was performed with the indicated primary antibodies at 4°C overnight. The membranes were then incubated with horseradish peroxidase (HRP)-conjugated secondary antibody (ImmunoWay, Plano, USA) at room temperature for 2 h. After incubating the membranes with ECL Plus Western Blotting Substrate (Thermo Fisher Scientific, Shanghai, China), immunolabeled proteins were detected using X-ray film or Chemiluminescent Imaging System (Tanon 5200, Shanghai, China).

For immunoprecipitation assay, cell lysates were harvested in 500 µL RIPA buffer supplemented with protease inhibitor, phosphatase inhibitor cocktails and 1 mM PMSF, followed by centrifugation at 15000 rpm for 15 min at 4°C. Protein concentration in the supernatant was quantified by the BCA Protein Assay Kit. The supernatant was incubated with specific primary antibody or control IgG at 4°C overnight, then further incubated with protein A/G magnetic beads (Biomake, Beijing, China) for 6 h. The immunoprecipitated pellet was washed with RIPA buffer at least three times, and proteins were eluted by boiling in SDS loading buffer. The eluted proteins (30 µg) were analyzed by SDS-PAGE and immunoblotting.

### Real-Time PCR Analyses

Total RNA was extracted using RNAiso Plus (TaKaRa, Kusatsu, Shiga, Japan) and then reverse-transcribed into cDNA using the PrimeScript RT reagent kit (TaKaRa, Kusatsu, Shiga, Japan) according to the manufacturer’s instructions. Prepared cDNA was then subjected to quantitative PCR analysis using 2×SYBR Green qPCR Master Mix (Promega, Shanghai, China). Real-time PCR assays were performed to quantify mRNA levels of human Survivin, Cyclin D1 and Prominin 1 (PROM1) genes. The comparative Ct method was used to analyze relative expression of genes. The data was presented as the fold change. The fold change was calculated as 2^-ΔΔCt^, where ΔΔCt = ΔCt_treated_ -ΔCt_control._ Ct is the cycle number at which fluorescence first exceeds the threshold. The ΔCt values from each target gene were obtained by subtracting the values for GAPDH Ct from the sample Ct. The primer pairs used for quantitative PCR amplification were as follows: Survivin sense, 5’-AGGACCACCGCATCTCTACAT-3’ and antisense, 5’-AAGTCTGGCTCGTTCTCAGTG-3’; Cyclin D1 sense, 5’-AATGACCCCGCACGATTTC-3’ and antisense, 5’-TCAGGTTCAGGCCTTGCAC-3’; PROM1 sense, 5’-AGGCTACTTTGAACATTATCTGC-3’ and antisense, 5’-GGCTTGTCATAACAGGATTGT-3’; GAPDH sense, 5’-CCAGAACATCATCCCTGCCTCTACT-3’ and antisense, 5’-GGTTTTTCTAGACGGCAGGTCAGGT.

### Chromatin Immunoprecipitation (ChIP) Assays

SW480 cells were treated with 240 nM SR3029 for 24 h, and ChIP assay was performed with ChIP-IT Express Enzymatic Chromatin Immunoprecipitation Kit (Sigma-Aldrich, Merck, USA) according to the manufacturer’s protocols. The eluted DNA was amplified by quantitative PCR analysis using an ABI Prism 7500 Real-Time PCR System (Applied Biosystems, Foster city, CA, USA). Primer sequences were shown as follows: Survivin sense, 5’-GCGTTCTTTGAAAGCAGT-3’ and antisense, 5’-ATCTGGCGGTTAATGGCG-3’; Axin2 sense, 5’-TCTGGTAGCATTATGGCCATCGCA-3’ and antisense, 5’-AAAGTCCTCCAAGCCCAAATTCCC-3’; The antibodies used were anti-β-catenin and anti-mouse IgG.

### Cell Viability Assays

SW480 and HCT116 cells were seeded in 96-well plates at a density of 5000 cells per well and cultured for 24 h. The cells were treated with SR3029 and MG149, alone or combined, at indicated concentrations for 48 h. Subsequently, 10 μL of MTT solution (5 mg/mL) was added into each well. The plate was further incubated for 4 h at 37°C and 100 μL of dimethyl sulfoxide (DMSO) was added to dissolve the insoluble formazan. The absorbance of the formazan solution was measured at 570 nm. Each treatment was performed in three replicates.

### BrdU Cell Proliferation Assays

SW480 and HCT116 cells were plated on 96-well plates at a density of 5000 cells per well and cultured for 24 h. The cells were treated with SR3029 and MG149, alone or combined, at indicated concentrations for 48 h. The BrdU incorporation assay was performed using the Cell Proliferation ELISA BrdU Colorimetric Kit (Roche, Basel, Switzerland) according to the manufacturer’s instructions. BrdU incorporation was detected by measuring the absorbance at 450 nm. Each treatment was performed in three replicates.

### Statistical Analyses

Statistical analyses were conducted using GraphPad Prism 7 software (La Jolla, CA, USA). The experiments were repeated three times, and data were exhibited as mean ± standard deviation (SD). The Student’s *t*-test was applied to determine the significance of difference between the two groups. One-way analysis of variance (ANOVA) with Dunn’s multiple comparisons test were utilized to compare the means of several groups. P<0.05 was defined as statistically significant.

## Results

### CK1δ/ϵ Increase β-Catenin-Mediated Transcription Through Regulating β-Catenin Acetylation

**Figure 1:**
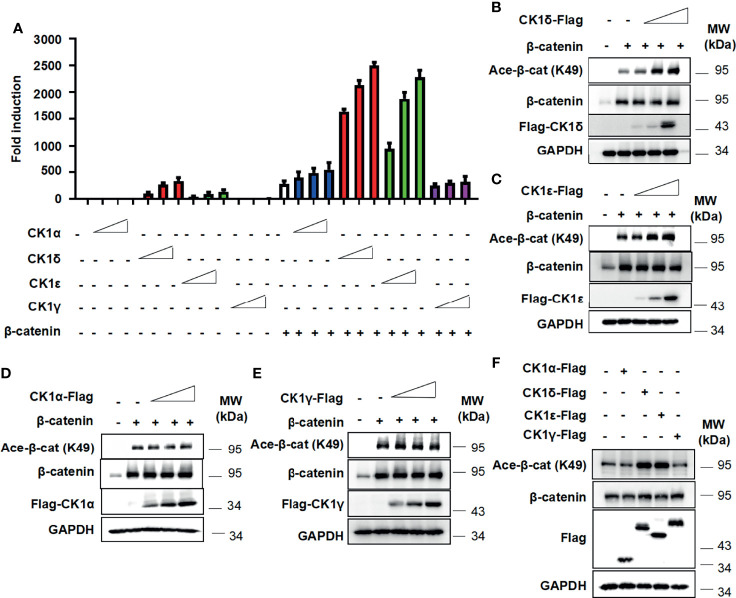
CK1δ/ϵ enhances β-catenin-mediated Wnt signaling and increases β-catenin acetylation at K49. **(A)** The SuperTopFlash reporter was transfected into HEK293T cells with β-catenin expression plasmid together with control vector or increasing amounts (10, 25, 50 ng) of expression plasmids encoding CK1α, CK1δ, CK1ϵ, and CK1γ, respectively. The luciferase values were normalized to β-gal activities. Each treatment was performed in four replicates. **(B–E)** HEK293T cells were transfected with empty vector or β-catenin along with increasing amounts (10, 25, 50 ng) of expression plasmids encoding CK1α, CK1δ, CK1ϵ and CK1γ, respectively. **(F)** HEK293T cells were transfected with 100 ng of control vector or expression plasmids encoding CK1α, CK1δ, CK1ϵ and CK1γ, respectively. Protein expression of total β-catenin, acetylated β-catenin at K49, Flag and GAPDH was measured by Western blotting.

To examine the effect of CK1 family members on β-catenin-mediated Wnt signaling, the Wnt-responsive reporter SuperTopFlash was transfected into HEK293T cells with expression vector encoding β-catenin, together with CK1α, CK1δ, CK1ϵ and CK1γ expression plasmids, respectively. As shown in **Figure 1A**, CK1δ or CK1ϵ dramatically enhanced β-catenin-mediated transcription in a dose-dependent manner, while CK1δ or CK1ϵ alone had a moderate effect on the SuperTopFlash reporter activity. Comparatively, CK1α or CK1γ exerted little effect on β-catenin-mediated activity **(** **Figure 1A** **)**. Since β-catenin can be acetylated at lysine 49 (K49), which is required for the activation of Wnt/β-catenin signaling (3, 5, 39, 40), we tested whether CK1 family members have any effect on the acetylation of β-catenin using an antibody against acetyl-K49 β-catenin. Our results showed that CK1δ or CK1ϵ significantly increased the K49 acetylation of exogenous β-catenin **(** **Figures 1B, C** **)**. In contrast, CK1α or CK1γ had little effect on the acetyl-K49 β-catenin level **(** **Figures 1D, E** **)**. Furthermore, we detected enhanced level of the K49 acetylation of endogenous β-catenin in HEK293T cells transfected with CK1δ or CK1ϵ, but not CK1α or CK1γ **(** **Figure 1F** **)**. These results revealed that CK1δ and CK1ϵ may increase β-catenin-mediated transcriptional activity through regulating β-catenin acetylation.

### CK1δ/ϵ Promote β-Catenin Acetylation *via* Recruiting Tip60

**Figure 2:**
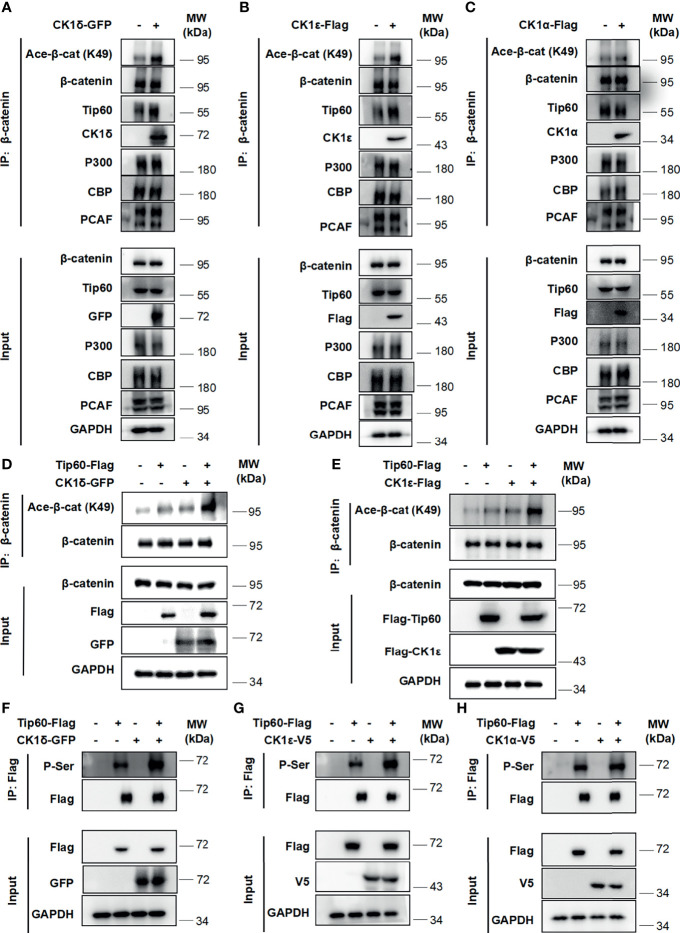
CK1δ/ϵ promotes β-catenin acetylation through enhancing the association of β-catenin with Tip60. **(A–C)** HEK293T cells were transfected with CK1δ-GFP, CK1ϵ-Flag and CK1α-Flag expression plasmids, and cell lysates were immunoprecipitated with anti-β-catenin agarose beads. The interaction between β-catenin and some histone acetyltransferases (Tip60, PCAF, p300, and CBP) was detected by immunoblotting. **(D, E)** HEK293T cells were transfected with control vector or Tip60-Flag expression plasmid in the presence or the absence of either CK1δ-Flag **(D)** or CK1ϵ-Flag **(E)** expression vector. The β-catenin protein was pulled down with anti-β-catenin agarose beads. The expression of β-catenin, acetylated β-catenin at K49, Flag-tagged proteins and GAPDH was detected by Western blotting. **(F–H)** HEK293T cells were transfected with control vector or Tip60-Flag expression plasmid in the presence or absence of either CK1δ-GFP **(F)** or CK1ϵ-V5 **(G)** or CK1α-V5 **(H)** expression plasmids. Whole cell lysates were immunoprecipitated with anti-Flag agarose beads. The expression of serine-phosphorylated Tip60, Tip60-Flag, CK1δ-GFP, CK1ϵ-V5, CK1α-V5 and GAPDH was measured by Western blotting. The expression of serine-phosphorylated Tip60 was detected by an anti-phospho-serine antibody.

Multiple histone acetyltransferases, including p300, CBP and PCAF, have been shown to acetylate β-catenin at different lysine residues, resulting in enhancing its transcriptional activity and up-regulating the expression of Wnt target genes (5–8). We then assessed whether CK1δ/ϵ could promote the interaction between β-catenin and some histone acetyltransferases. A coimmunoprecipitation assay was performed using an anti-β-catenin antibody in HEK293T cells that were transiently transfected with GFP-tagged CK1δ, Flag-tagged CK1ϵ or CK1α. The results showed that the expression of CK1δ or CK1ϵ did not affect the interaction between β-catenin and p300 or CBP or PCAF. Surprisingly, CK1δ or CK1ϵ could dramatically enhance the binding of β-catenin to Tip60 **(** **Figures 2A, B** **)**, while CK1α had little effect on the interaction between β-catenin and Tip60 **(** **Figure 2C** **)**. To further confirm the effect of CK1δ/ϵ and Tip60 on β-catenin acetylation, HEK293T cells were transfected with Tip60-Flag expression vector along with expression plasmids for GFP-CK1δ or Flag-CK1ϵ. Cell lysates were extracted from the transfected cells and immunoprecipitated with an anti-β-catenin antibody. Western blotting analysis showed that simultaneous expression of Tip60 and CK1δ or CK1ϵ markedly increased the acetylation of β-catenin at K49 compared to cells expressing Tip60 or CK1δ or CK1ϵ alone **(** **Figures 2D, E** **)**. To test whether CK1δ or CK1ϵ could phosphorylate Tip60, HEK293T cells were transfected with Tip60-Flag expression plasmid along with expression vectors for CK1δ or CK1ϵ or CK1α, respectively. Total cell extracts were used for affinity purification by anti-Flag M2 agarose. We observed that anti-Flag M2 agarose could pull down Tip60-Flag, and the presence of CK1δ or CK1ϵ elevated the level of phosphorylated Tip60, detected by a pan phospho-serine antibody, while the expression of CK1α did not affect Tip60 phosphorylation **(** **Figures 2F–H** **)**. These results suggest that the binding of CK1δ/ϵ to Tip60 induced Tip60 phosphorylation and facilitated the recruitment of Tip60 to β-catenin complex, resulting in the acetylation of β-catenin.

### Tip60 Significantly Enhances the Transcriptional Activity Induced by CK1δ/ϵ or/and β-Catenin

**Figure 3:**
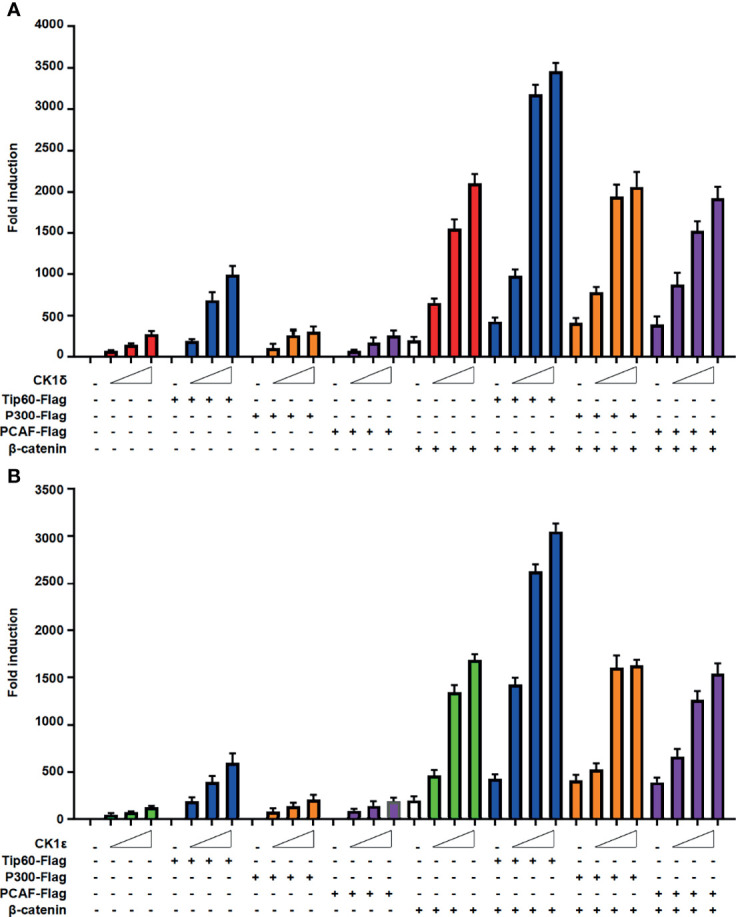
Tip60 enhances the transcriptional activity induced by CK1δ/ϵ or/and β-catenin. **(A, B)** The SuperTopFlash reporter was transfected into HEK293T cells with control vector or increasing amounts of CK1δ **(A)** or CK1ϵ **(B)** expression plasmids together with expression vectors encoding Tip60-Flag, p300-Flag and PCAF-Flag with or without β-catenin expression vector. The luciferase values were normalized to β-gal activities. Each treatment was performed in four replicates.

We next evaluated the effect of CK1δ/ϵ, β-catenin and Tip60, alone or combined, on the Wnt signaling pathway. The SuperTopFlash reporter was transfected into HEK293T cells together with CK1δ/ϵ, β-catenin and Tip60 expression plasmids, alone or combined, as indicated in **Figure 3**. Overexpression of Tip60 significantly enhanced the SuperTopFlash reporter activity induced by CK1δ or CK1ϵ or β-catenin or CK1δ/β-catenin or CK1ϵ/β-catenin, while Tip60 alone had no any effect on the transcription of SuperTopFlash reporter **(** **Figures 3A, B** **)**. Notably, p300 or PCAF had little effect on CK1δ/ϵ-mediated transcriptional activity **(** **Figures 3A, B** **)**. These results indicated that Tip60 could positively modulate Wnt signaling in the presence of CK1δ/ϵ or/and β-catenin.

### The CK1δ/CK1ϵ/β-Catenin/Tip60 Complex Exists in Colon Cancer Cells and β-Catenin Interacts With Tip60 in a CK1δ/CK1ϵ-Dependent Manner

**Figure 4:**
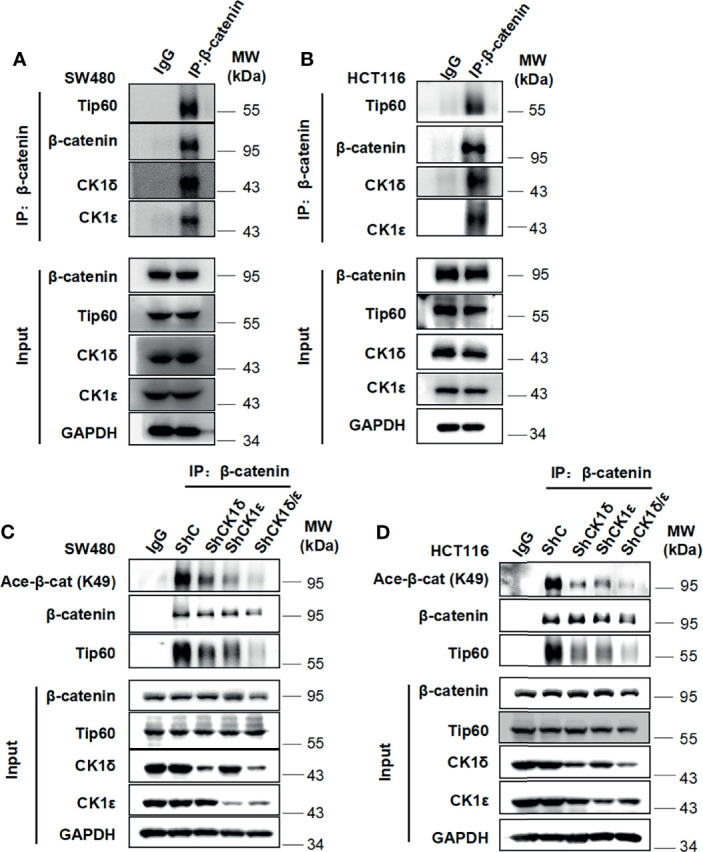
The CK1δ/CK1ϵ/β-catenin/Tip60 complex is detected in colon cancer cells and β-catenin interacts with Tip60 in a CK1δ/CK1ϵ-dependent manner. **(A, B)** Cell lysates from SW480 **(A)** or HCT116 **(B)** cells were immunoprecipitated with normal IgG control or anti-β-catenin agarose beads, Immunoblot analysis was performed to detect the interaction among β-catenin, Tip60 and CK1δ/ϵ using the indicated antibodies. **(C, D)** SW480 **(C)** or HCT116 **(D)** cells were infected with shNC, shCK1δ-2, shCK1ϵ-2, shCK1δ/ϵ-2 lentivirus, then cells were lysed and subjected to immunoprecipitation with anti-β-catenin agarose beads. Immunoblotting was performed using the indicated antibodies.

Considering that CK1δ and CK1ϵ are highly expressed in colon cancer, which is involved in advanced progression and poor prognosis (34, 41, 42), two colon cancer cell lines SW480 and HCT116 were employed to examine the interaction among CK1δ/ϵ, β-catenin and Tip60. These two cell lines have different types of mutations, SW480 cells harboring truncated mutation of APC and HCT116 cells with S45 mutation of β-catenin. The APC and β-catenin mutations are the most frequently occurred mutation types in components of the Wnt signaling pathway in colon cancer (43–45). The result of immunoprecipitation assay showed that the endogenous CK1δ, CK1ϵ, β-catenin and Tip60 proteins were immunoprecipitated with an anti-β-catenin antibody in SW480 and HCT116 cells **(** **Figures 4A, B** **)**, suggesting that the CK1δ/CK1ϵ/β-catenin/Tip60 complex existed in colon cancer cells. To explore the effect of CK1δ/ϵ on the interaction between β-catenin and Tip60, lentivirus-mediated shRNAs were used to knockdown the expression of CK1δ and CK1ϵ, either alone or in combination, in colon cancer cells. Simultaneous knockdown of CK1δ and CK1ϵ dramatically attenuated the association of β-catenin with Tip60 in SW480 and HCT116 cells **(** **Figures 4C, D** **)**. These results suggest that β-catenin may interact with Tip60 in a CK1δ/CK1ϵ-dependent manner.

### Knockdown of CK1δ/ϵ or Treatment With CK1 Inhibitors Downregulate the Level of β-Catenin Acetylation at K49 and Inhibit the Viability and Proliferation in Colon Cancer Cells

**Figure 5:**
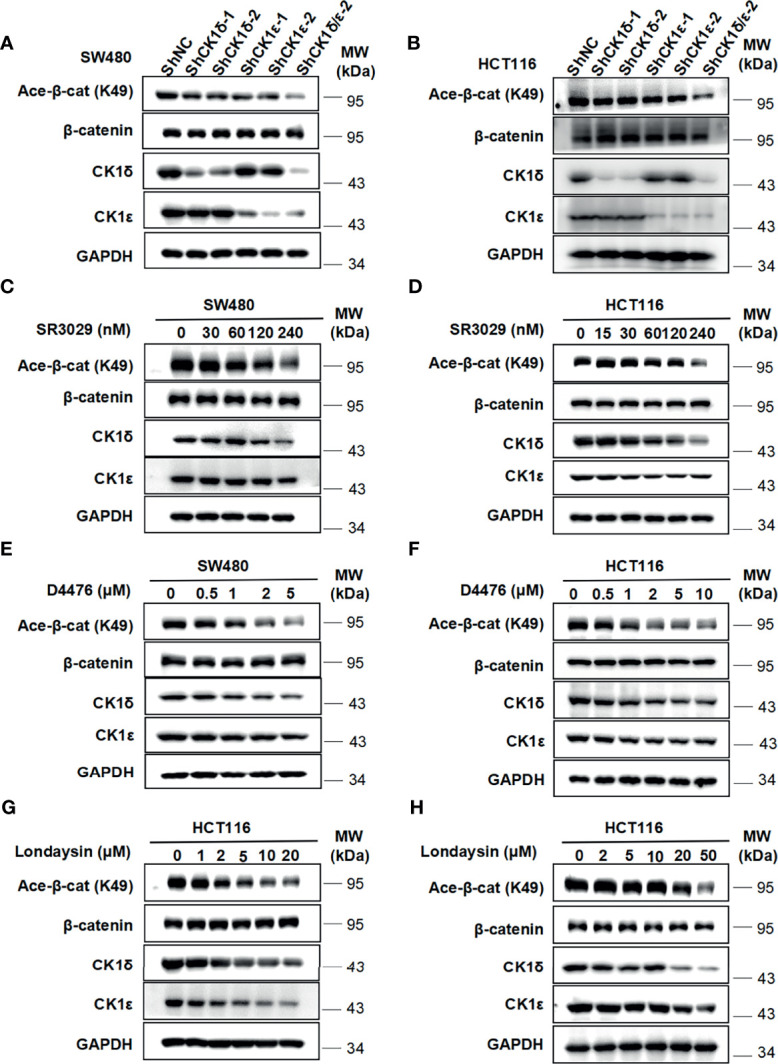
Depletion of CK1δ/ϵ or treatment with CK1 inhibitors downregulate the level of β-catenin acetylation at K49 in colon cancer cells. **(A, B)** The expression of endogenous CK1δ and CK1ϵ was knocked down by infecting SW480 **(A)** and HCT116 **(B)** cells with shNC, shCK1δ-1, shCK1δ-2, shCK1ϵ-1, shCK1ϵ-2 and shCK1δ/ϵ-2 lentivirus. The levels of β-catenin, acetylated β-catenin at K49, CK1δ, CK1ϵ and GAPDH were detected by immunoblotting. **(C, D)** SW480 **(C)** and HCT116 **(D)** cells were serum-starved for 12 h and subsequently treated with the indicated amounts of SR3029 for 12 h Cell lysates were subjected to immunoblotting with the indicated antibodies. **(E, F)** Similar to panel C and D except that the indicated concentrations of D4476 were used. **(G, H)** Similar to panel **(C, D)** except that the indicated concentrations of longdaysin were used.

We next check the effect of CK1δ/ϵ on β-catenin acetylation at K49 in colon cancer cells. As shown in **Figures 5A, B**, the level of the acetylation of β-catenin at K49 was dramatically reduced in two colon cancer cell lines (SW480 and HCT116) with knockdown of CK1δ and CK1ϵ simultaneously **(** **Figures 5A, B** **)**. Moreover, treatment with small molecular CK1 inhibitors (SR3029, D4476 and longdaysin) markedly decreased the K49 acetylation of β-catenin in SW480 and HCT116 cells, while depletion of CK1δ/ϵ or treatment with small molecular CK1 inhibitors had little effect on the expression of Tip60 and β-catenin **(** **Figures 5C–H** and **Figures S1A–H** **)**. In contrast, proteasome inhibitor MG132 increased the expression of acetylated β-catenin and total β-catenin in SW480 and HCT116 cells (**Figures S2A, B**). Consistently, treatment with the protein synthesis inhibitor CHX did not alter the kinetics of β-catenin degradation in CK1δ/ϵ-knockdown SW480 cells (**Figure S2C**). However, we detected significantly decreased level of β-catenin acetylation at K49 in CK1δ/ϵ-knockdown cells (**Figure S2C**). These results indicated that CK1δ and CK1ϵ could enhance the acetylation of β-catenin, but had little effect on its stability in colon cancer cells.

We further assessed whether the inhibition of CK1δ/ϵ activity by SR3029 had any effect on β‐catenin binding to the Wnt target gene promoter. The ChIP assay was performed to detect β‐catenin binding to the promoters of two known Wnt target genes, Survivin and Axin2, in SW480 cells. Our results showed that SR3029 treatment significantly reduced β‐catenin binding to the promoters of Survivin and Axin2 (**Figures S3A, B**). We also examined the effect of CK1δ and CK1ϵ on colon cancer cell viability and proliferation. Knockdown of either CK1δ or CK1ϵ decreased the viability and proliferation in SW480 and HCT116 cells. Simultaneous knockdown of CK1δ and CK1ϵ exerted greater inhibition of viability and proliferation in both cells (**Figures S4A–D**). Taken together, these results illustrated that silencing of CK1δ/1ϵ or treatment with CK1 inhibitor could reduce the β-catenin acetylation and suppress β‐catenin binding to the promoters of Wnt target genes, resulting in the inhibition of colon cancer cell viability and proliferation.

### Silencing Tip60 or Treatment With Tip60 Inhibitors Downregulate the Level of β-Catenin Acetylation at K49 in Colon Cancer Cells

**Figure 6:**
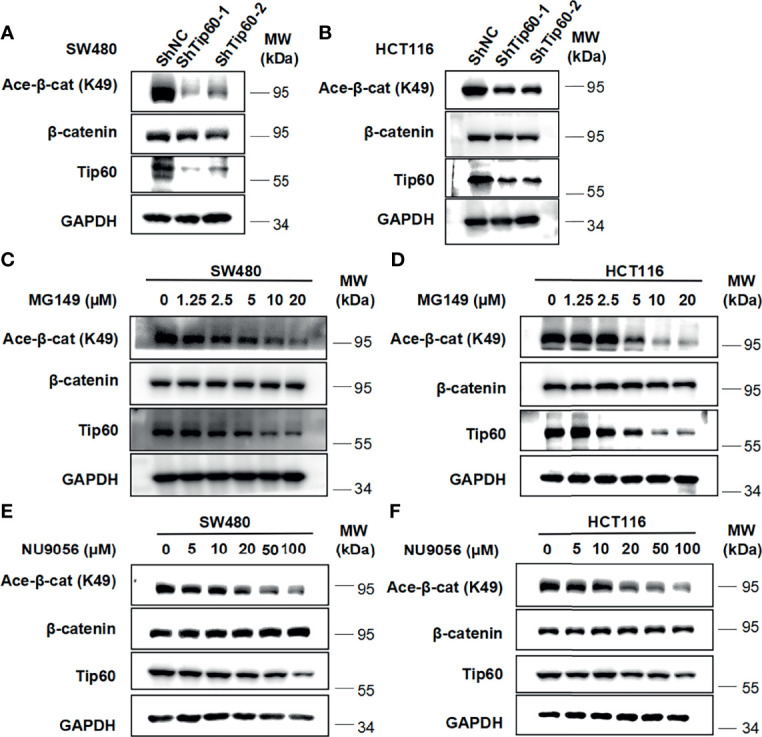
Silencing Tip60 or treatment with Tip60 inhibitors downregulate the level of β-catenin acetylation at K49 in colon cancer cells. **(A, B)** SW480 **(A)** and HCT116 **(B)** cells were infected with shNC, shTip60-1 and shTip60-2 lentivirus, respectively. The expression of β-catenin, acetylated β-catenin at K49, CK1δ, CK1ϵ and GAPDH was measured by immunoblotting. **(C, D)** SW480 **(C)** and HCT116 **(D)** cells were serum-starved for 12 h and subsequently treated with the indicated amounts of MG149 for 12 h Cell lysates were subjected to immunoblotting with the indicated antibodies. **(E, F)** Similar to panel **(C, D)** except that the indicated concentrations of NU9056 were used.

To determine whether Tip60 is able to regulate the acetylation of β-catenin, we silenced the expression of Tip60 using lentivirus-mediated shRNAs in colon cancer cells. Depletion of Tip60 downregulated the level of β-catenin acetylation at K49 without influencing total β-catenin expression in SW480 and HCT116 cells **(** **Figures 6A, B** **)**. Two small molecular Tip60 inhibitors MG149 and NU9056 were used to block the activity of Tip60 in SW480 and HCT116 cells. The K49 acetylation of β-catenin was significantly reduced upon treatment with either MG149 or NU9056 **(** **Figures 6C–F** **)**. Collectively, our results illustrated that Tip60 could regulate β-catenin acetylation in colon cancer cells.

### Combined Treatment With SR3029 and MG149 Has More Potent Effect on β-Catenin Acetylation, the Transcription of Wnt Target Genes, Cell Viability and Proliferation in Colon Cancer Cells

**Figure 7:**
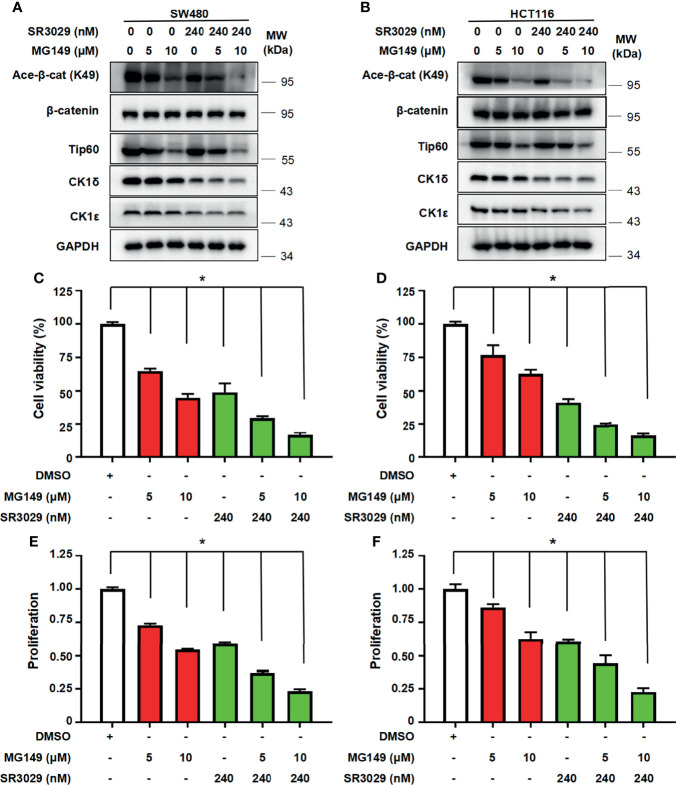
Combined treatment with SR3029 and MG149 exhibits more potent effect on β-catenin acetylation, cell viability and proliferation in colon cancer cells. **(A, B)** SW480 **(A)** and HCT116 **(B)** cells were serum-starved for 12 h and subsequently treated with MG149 (5 and 10 μM) alone or combined with 240 nM SR3029 for 12 h The protein levels of β-catenin, acetylated β-catenin at K49, Tip60, CK1δ, CK1ϵ and GAPDH were detected by immunoblotting. **(C–F)** SW480 **(C, E)** and HCT116 **(D, F)** cells were serum-starved for 12 h and subsequently treated with MG149 (5 and 10 μM) alone or combined with 240 nM SR3029. After 48 h of treatment, MTT assay was used to detect cell viability **(C, D)**. Cell proliferation was detected using BrdU incorporation assay **(E, F)**. The data from three independent experiments are presented (n=3). Values shown are means ± SD. *P<0.05, significantly different from the vehicle control; one-way ANOVA followed by Dunnett’s test **(C–F)**.

To assess the potential consequence of combined inhibition of CK1δ/ϵ and Tip60 activities, we tested the effect of combined treatment with CK1δ/ϵ inhibitor SR3029 and Tip60 inhibitor MG149 on β-catenin acetylation, the transcription of Wnt target genes, cell viability and proliferation in colon cancer cells. SW480 and HCT116 cells were treated with SR3029 (240 nM) and MG149 (5 and 10 μM), alone or combined. Our results showed that combined treatment markedly downregulated the level of β-catenin acetylation at K49 **(** **Figures 7A, B** **)** and the transcription of Wnt target genes Survivin, Cyclin D1 and PROM1 **(** **Figures S5A–F** **)** compared with either drug alone. Furthermore, combined treatment of SR3029 and MG149 was more effective than either drug alone in inhibiting viability and proliferation in SW480 and HCT116 cells **(** **Figures 7C–F** **)**. These results revealed that combined inhibition of CK1δ/ϵ and Tip60 activities exert a more profound inhibitory effect on β-catenin acetylation, the transcription of Wnt target genes, the viability and proliferation in colon cancer cells.

## Discussion

Aberrant activation of canonical Wnt/β-catenin signaling plays a crucial role in proliferation, cellular stemness and chemoresistance in colon cancer (43). Mutations in the APC, β-catenin and RNF43 genes result in the abnormal accumulation of β-catenin and upregulation of Wnt target genes (43–46). Moreover, CK1δ and CK1ϵ have been shown to be highly expressed in colon cancer tissues. The upregulation of CK1δ and CK1ϵ were closely associated with advanced progression and poorer prognosis of colon cancer (34, 41, 42). So far, the molecular mechanism underlying CK1δ/ϵ-mediated downstream events in the Wnt/β-catenin signaling cascade remain unclear. In the present study, we demonstrated that CK1δ/ϵ increased β-catenin-mediated transcription *via* modulating the acetylation of β-catenin at K49. The interaction of CK1δ/ϵ with Tip60 recruited Tip60 to β-catenin complex, leading to β-catenin acetylation and the activation of Wnt signaling. Our results revealed a novel molecular mechanism by which CK1δ/ϵ enhances β-catenin-mediated transcription in colon cancer.

Previous studies have reported that different CK1 family members could exert a coordinated action in the activation of Wnt signaling (26). CK1α has been shown to phosphorylate β-catenin at S45, which is required for subsequent GSK-3β phosphorylation and degradation of β-catenin (26–28). CK1δ and CK1ϵ could positively regulate the Wnt/β-catenin pathway by acting on multiple targets, such as DVL, LRP6 and AES (32–34, 47, 48). In this study, our results demonstrated that CK1δ/ϵ could interact with and phosphorylate Tip60, promote the association of β-catenin with Tip60, and lead to the acetylation of β-catenin at K49. Simultaneous knockdown of CK1δ and CK1ϵ, or treatment with chemical CK1 inhibitors caused the downregulation of β-catenin acetylation at K49 in colon cancer cells. As expected, we observed that the expression of CK1α or CK1δ or CK1ϵ increased the phosphorylation of endogenous or exogenous β-catenin at S45 **(** **Figures S6A, B** **)**. However, CK1α had little effect on Tip60 phosphorylation and the interaction between β-catenin with Tip60. These results suggest that CK1δ/ϵ-mediated phosphorylation of β-catenin at S45 may not be related to the positive regulatory function of CK1δ/ϵ in the Wnt pathway.

Increasing evidence shows that the acetylation of β-catenin has been implicated in regulating its stability, subcellular location, specific interactions, and transactivational activity (5, 6, 8, 49). The β-catenin protein has been reported to be acetylated by CBP at K49, which is frequently found mutated in multiple cancers (5). Levy et al. showed that the acetylation of β-catenin at K345 by p300 increased the affinity of β-catenin for TCF4 (6). PCAF has been shown to acetylate β-catenin at K19 and 49, improving the stability and transcriptional activity of β-catenin (7). Recently, cell-cycle related and expression-elevated protein (CREPT) was identified as a potential oncogene in colorectal cancer. CREPT could enhance the association of p300 with β-catenin, thus promoting p300-mediated β-catenin acetylation and stabilization (50). Li et al. reported that block of proliferation 1 (BOP1) could increase Wnt/β-catenin signaling by enhancing CBP recruitment to β-catenin, thus promoting β-catenin acetylation and activation (51). A recent study showed that Bcl-3 could enhance the Wnt signaling cascade by maintaining the acetylation of β-catenin at K 49 in colorectal cancer (40). In our study, we provided several lines of evidence to demonstrate that Tip60 could acetylate β-catenin at K49 in a CK1δ/ϵ-dependent manner. First, CK1δ or CK1ϵ significantly enhanced the association of β-catenin with Tip60, but not p300 or CBP or PCAF; Second, CK1δ/ϵ could bind to Tip60 and increased the phosphorylation of Tip60. Future studies are needed to identify specific phosphorylation sites of Tip60 by CK1δ/ϵ; Third, the CK1δ/CK1ϵ/β-catenin/Tip60 complex was observed in colon cancer cells, and simultaneous knockdown of CK1δ and CK1ϵ markedly attenuated the association of β-catenin with Tip60; Fourth, knockdown of CK1δ/ϵ or treatment with CK1 inhibitors downregulated the level of β-catenin acetylation at K49 in colon cancer cells. Silencing Tip60 or treatment with Tip60 inhibitors also decrease the level of β-catenin acetylation at K49; Fifth, combined treatment with CK1 inhibitor SR3029 and Tip60 inhibitor MG149 had more potent effect on downregulation of β-catenin acetylation and Wnt target genes. Finally, Tip60 significantly enhanced the SuperTopFlash reporter activity induced by CK1δ/ϵ or/and β-catenin. Taken together, our results showed that CK1δ/ϵ could increase the interaction between β-catenin and Tip60, facilitating the recruitment of Tip60 to β-catenin complex, resulting in the acetylation of β-catenin at K49 and promoting of β-catenin-mediated transcriptional activity. However, we could not check the acetylation status of other lysine residues of β-catenin due to the lack of suitable antibodies. Future studies are needed to examine whether Tip60 also acetylates other lysine residues of β-catenin in a CK1δ/ϵ-dependent fashion.

In conclusion, our study demonstrated that CK1δ/ϵ could increase the transcriptional activity of β-catenin through regulating β-catenin acetylation at K49. CK1δ/ϵ potentiated the association of β-catenin with Tip60, and facilitated the recruitment of Tip60 to β-catenin complex, thereby leading to the acetylation of β-catenin at K49. Blockade of CK1δ/ϵ or/and Tip60 downregulated β-catenin acetylation and the transcription of Wnt target genes, resulting in growth inhibition of colon cancer cells. Our study define a novel CK1δ/ϵ-β-catenin-Tip60 axis which is implicated in modulation of β-catenin-mediated transcription.

## Data Availability Statement

The original contributions presented in the study are included in the article/**Supplementary Material**. Further inquiries can be directed to the corresponding author.

## Author Contributions

JN and DL developed the concept and designed this work. JN, QS, ZS, LT, YT, SS, and HL performed the experiments, carried out the data acquisition. JN, QS, ZS, VX, SL, XC, and DL performed data analysis. JN, QS, and DL edited and revised the manuscript. DL supervised this study. All authors read and approved this manuscript.

## Funding

This work was supported by the National Natural Science Foundation of China (31970739 and 81802662), the Natural Science Foundation of Guangdong Province (2020A1515010340 and 2020A1515010543), the Shenzhen Key Basic Research Program (JCYJ20200109105001821), the Shenzhen Natural Science Fund (the Stable Support Plan Program) (20200826134656001).

## Conflict of Interest

The authors declare that the research was conducted in the absence of any commercial or financial relationships that could be construed as a potential conflict of interest.

## Publisher’s Note

All claims expressed in this article are solely those of the authors and do not necessarily represent those of their affiliated organizations, or those of the publisher, the editors and the reviewers. Any product that may be evaluated in this article, or claim that may be made by its manufacturer, is not guaranteed or endorsed by the publisher.
