# Supplementary material for: The CK1δ/ϵ-Tip60 Axis Enhances Wnt/β-Catenin Signaling via Regulating β-Catenin Acetylation in Colon Cancer
Source: Front Oncol. 2022 Apr 12;12:844477. doi: 10.3389/fonc.2022.844477 (PMC9039669; doi:10.3389/fonc.2022.844477)
Supplement: Supplementary file 1 [file DataSheet_1.docx]

**Supplementary materials**

**The CK1δ/ε-Tip60 axis enhances Wnt/β-catenin signaling via regulating β-catenin acetylation in colon cancer**

**Jiong Ning^1 2^†, Qi Sun^1^†, Zijie Su^1 3^, Lifeng Tan^1^, Yun Tang^1^, Sapna Sayed^1^, Huan Li^1^, Vivian Weiwen Xue^1^, Shanshan Liu^1^, Xianxiong Chen^1^ and Desheng Lu^1 2^***

^1^ Guangdong Provincial Key Laboratory of Regional Immunity and Diseases, International Cancer Center, Department of Pharmacology, Shenzhen University Health Science Center, Shenzhen, Guangdong 518055, China

^2^ Shenzhen University-Friedrich Schiller Universität Jena Joint PhD Program in Biomedical Sciences, Shenzhen University School of Medicine, Shenzhen, Guangdong 518055, China.

^3^ Department of Research, The Affiliated Tumor Hospital of Guangxi Medical University, Nanning, Guangxi 530021, China

**†**These authors contributed equally to this work.

***** Corresponding author: Dr. Desheng Lu, e-mail: [delu@szu.edu.cn](mailto:delu@szu.edu.cn).

**
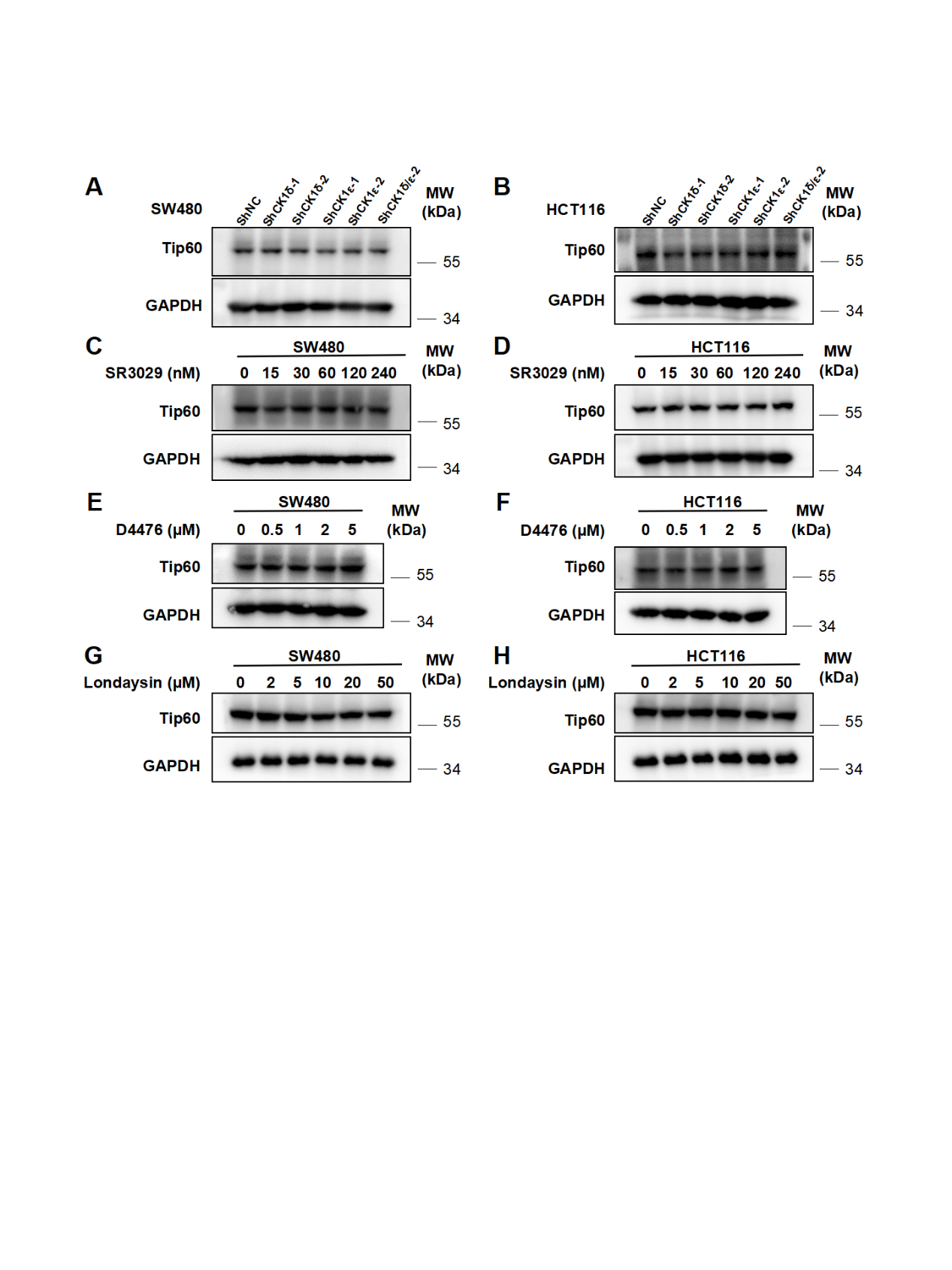
**

**Supplementary Figure 1.** Depletion of CK1δ/ε or treatment with CK1 inhibitors have little effect on the level of Tip60 in colon cancer cells. **(A, B)** The expression of endogenous CK1δ and CK1ε was knocked down by infecting SW480 **(A)** and HCT116 **(B)** cells with shNC, shCK1δ-1, shCK1δ-2, shCK1ε-1, shCK1ε-2 and shCK1δ/ε-2 lentivirus. The levels of Tip60 and GAPDH were detected by immunoblotting. **(C, D)** SW480 **(C)** and HCT116 **(D)** cells were serum-starved for 12 h and subsequently treated with the indicated amounts of SR3029 for 12 h. Cell lysates were subjected to immunoblotting with the indicated antibodies. **(E, F)** Similar to panel C and D except that the indicated concentrations of D4476 were used. **(G, H)** Similar to panel C and D except that the indicated concentrations of longdaysin were used.


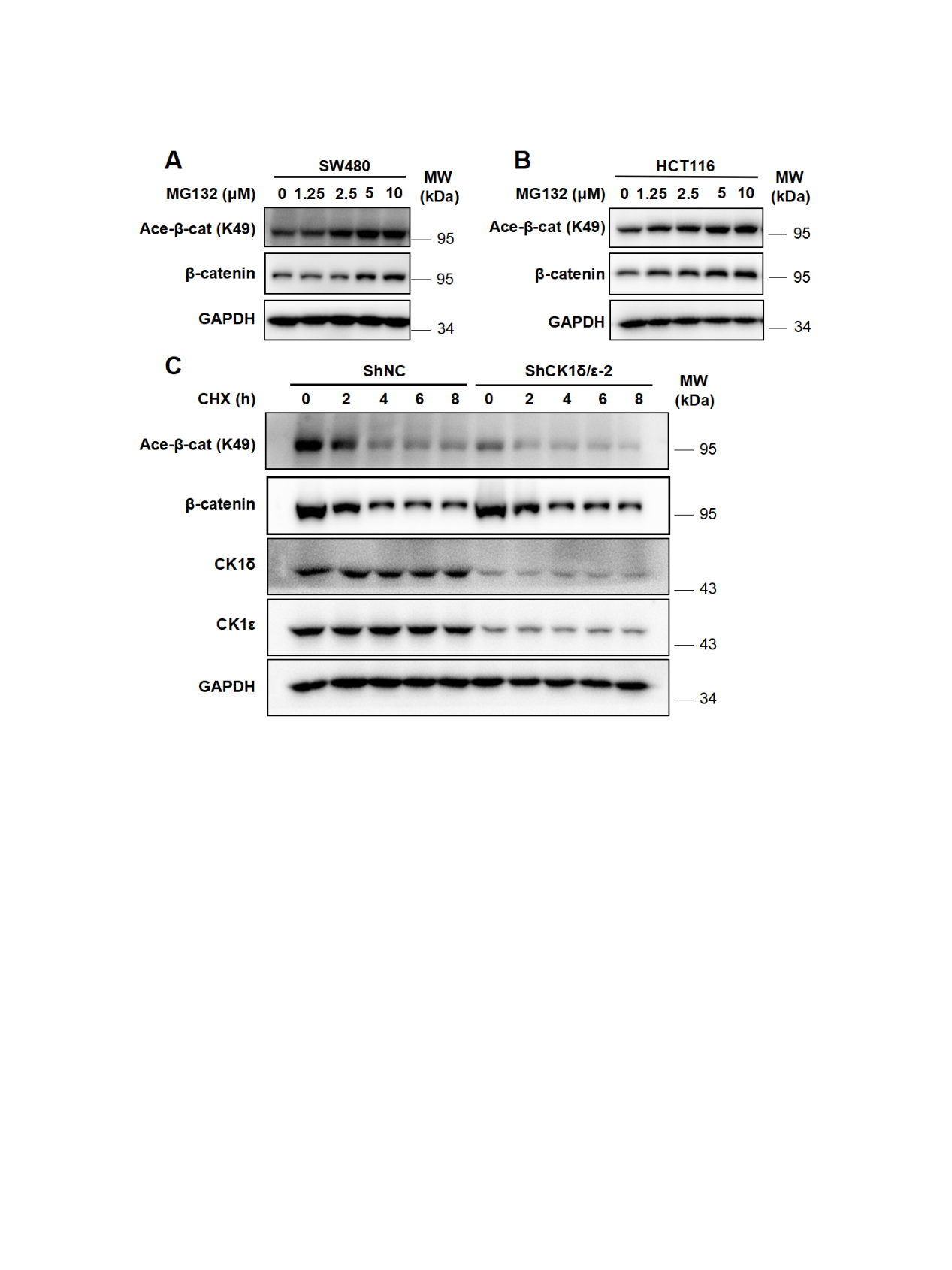


**Supplementary Figure 2.** The stability of β-catenin is mediated by the proteasome, but not by CK1δ/ε. **(A, B)** SW480 and HCT116 cells were treated with the indicated amounts of MG132 for 6 h, the levels of β-catenin, acetylated β-catenin at K49 and GAPDH were detected by immunoblotting.**(C)** SW480 cells were infected with shNC and shCK1δ/ε-2 lentivirus. The CK1δ/ε-knockdown cells and their parental control cells were treated with 50 μg/mL CHX for the indicated times. The levels of β-catenin, acetylated β-catenin at K49, CK1δ, CK1ε and GAPDH were measured by immunoblotting.


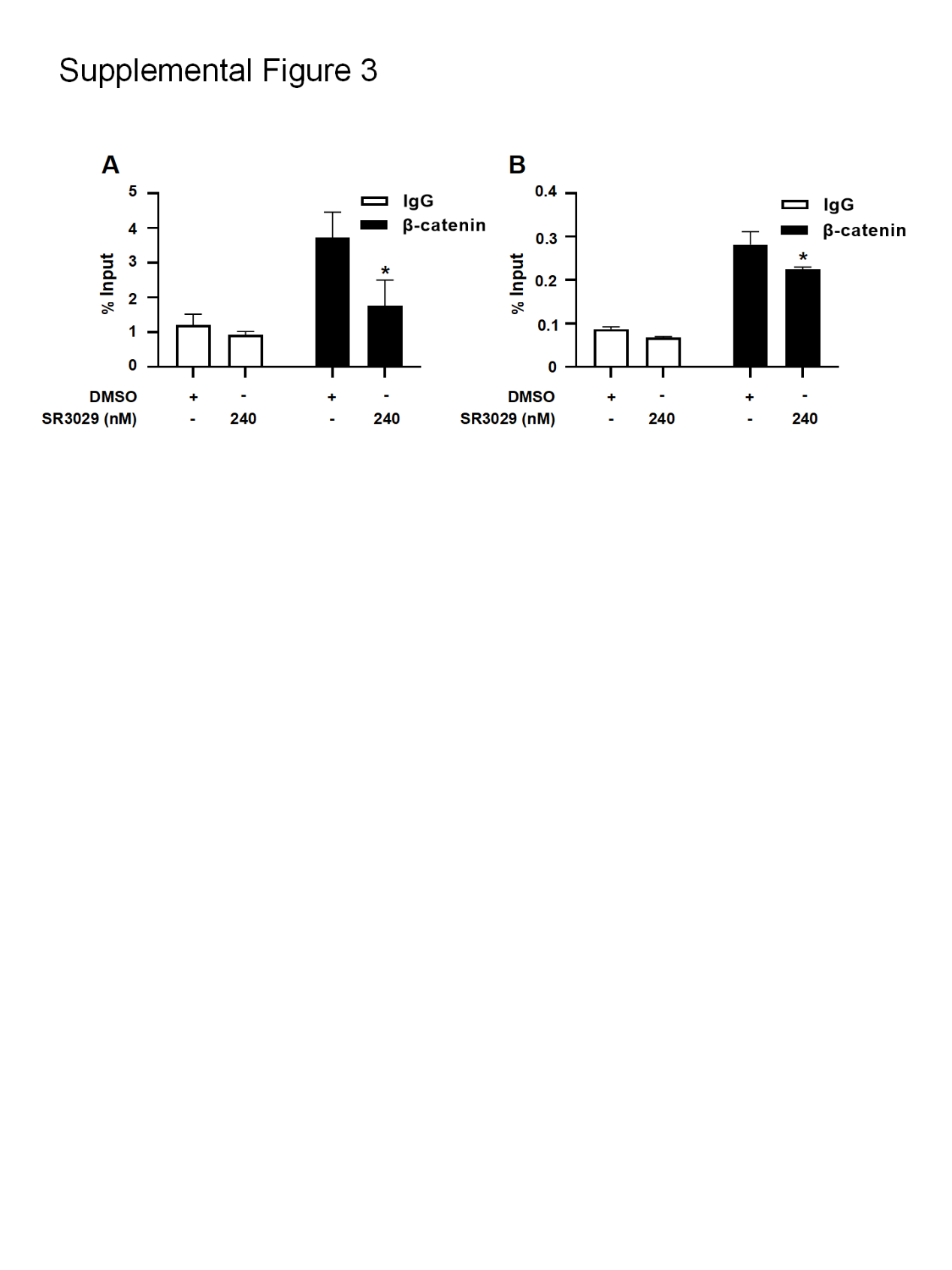


**Supplementary Figure 3.** SR3029 interferes with the recruitment of β-catenin to the promoters of Survivin and Axin2. SW480 cells were treated with 240 nM SR3029 for 12 h, and a ChIP assay was performed using the control IgG or anti-β-catenin. Real-time PCR analysis was used to detect the binding of β-catenin to the Survivin **(A)** and Axin2 **(B)** promoters. The data from three independent experiments are presented (n=3). Values shown are means +SD. *P<0.05, significantly different from the vehicle control; one-way ANOVA followed by Dunnett’s test.


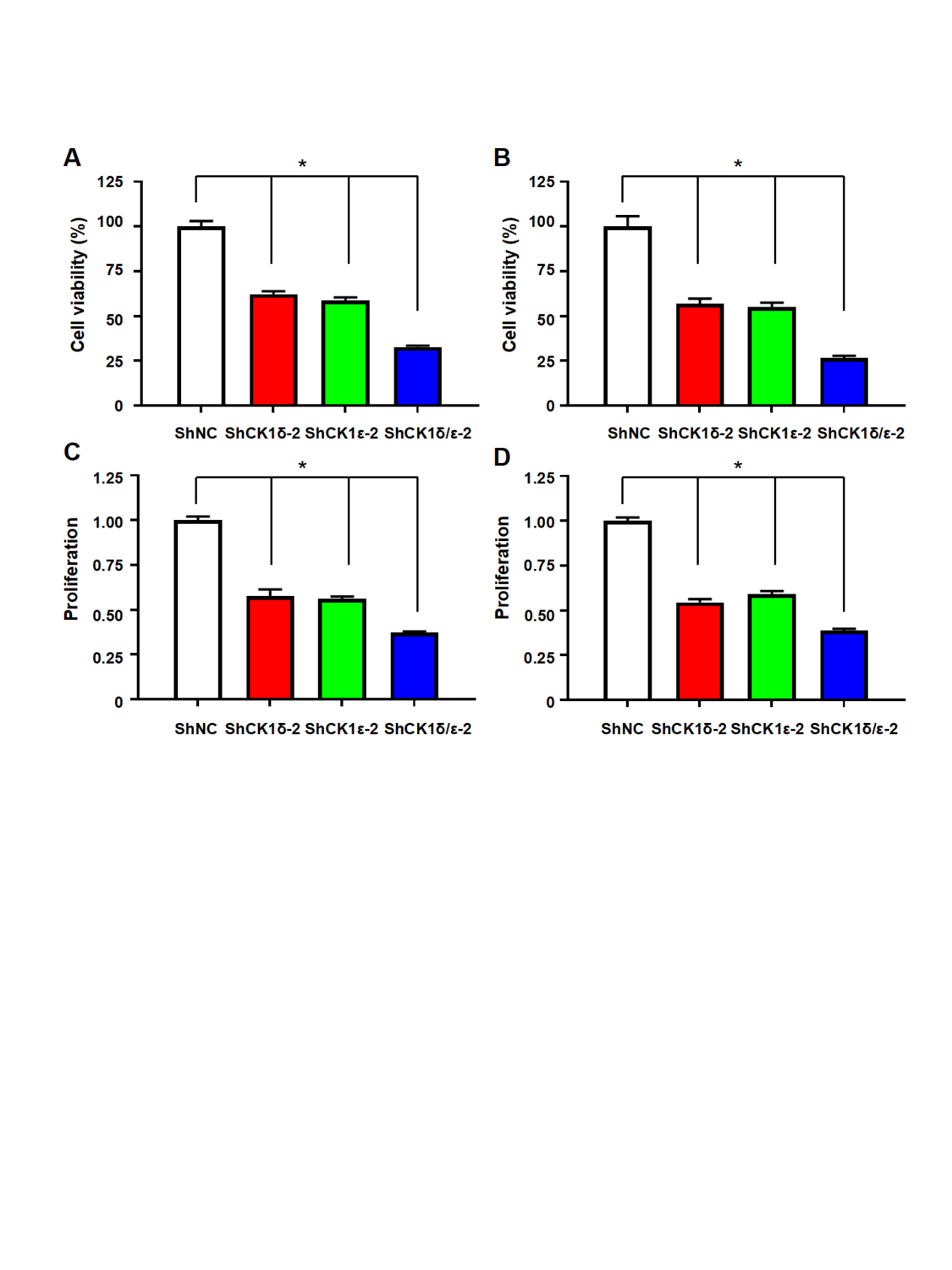


**Supplementary Figure 4.** Knockdown CK1δ/εresults in decreased viability and proliferation incolon cancer cells. SW480 and HCT116 cells were infected with lentivirus containing control shRNA (shNC) or shRNAs targeting CK1δ and CK1ε (shCK1). Then cells were seeded at 1×10^3^ cells/well in 96-well plates. After 48 h culture, cell viability was detected by MTT assay **(A, B),** and cell proliferation was measured by BrdU cell proliferation assay **(C, D)**. The data from three independent experiments are presented (n=3). Values shown are means +SD. *P<0.05, significantly different from the vehicle control; one-way ANOVA followed by Dunnett’s test.


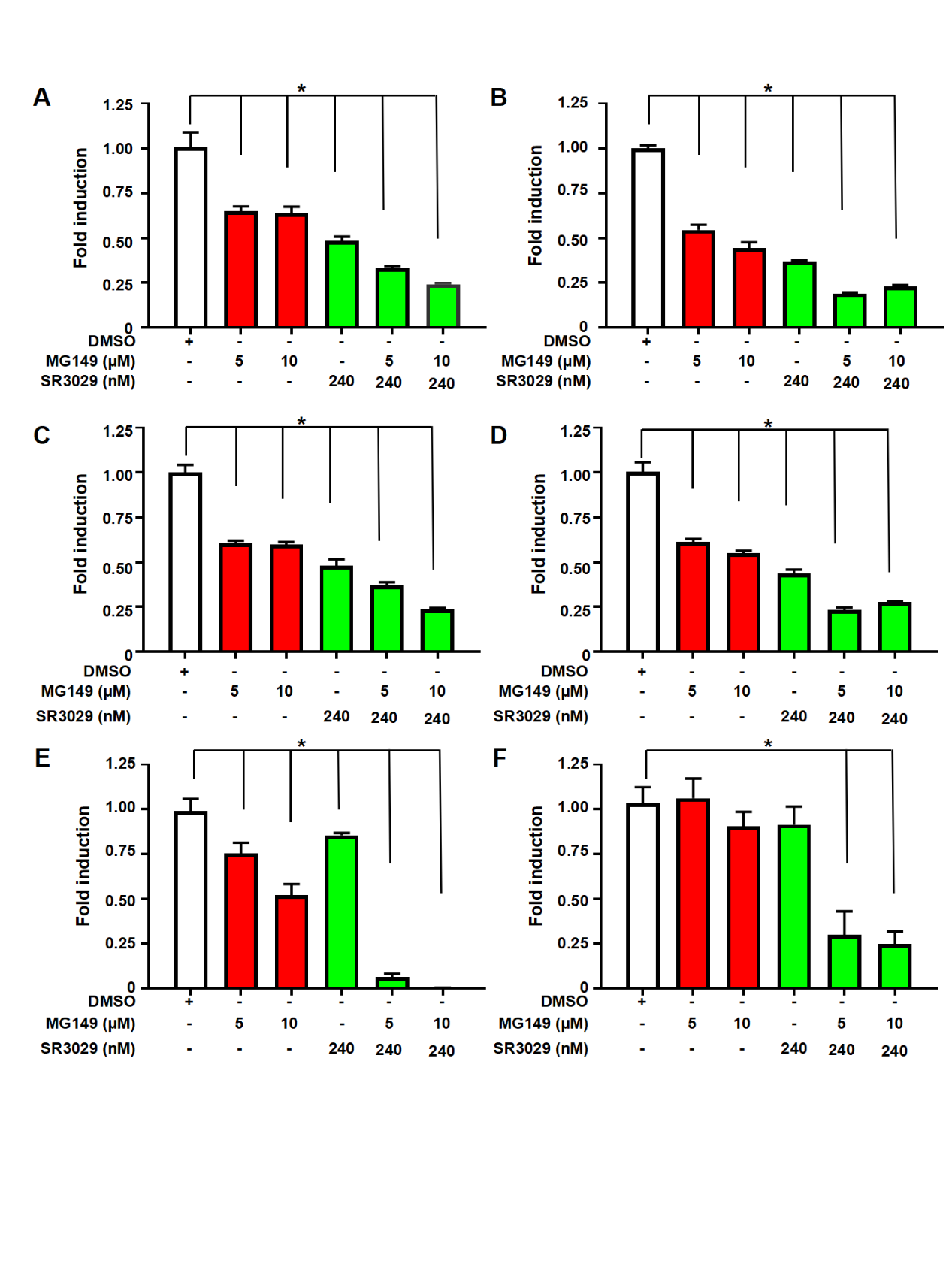


**Supplementary Figure 5.** MG149 alone or combined with SR3029 inhibit the transcriptional activity of Wnt target genes in colon cancer cells. **(A-F)** SW480 **(A, C, E)** and HCT116 **(B, D, F)** cells were serum-starved for 12 h and subsequently incubated with MG149 (5 and 10 μM) alone or combined with 240 nM SR3029 for 12 h. Total RNA was extracted, and real-time PCR analysis was used to detect the mRNA expression of Survivin **(A, B)**, Cyclin D1 **(C, D)** and PROM1 **(E, F)**. The data from three independent experiments are presented (n=3). Values shown are means +SD. *P<0.05, significantly different from the vehicle control; one-way ANOVA followed by Dunnett’s test.


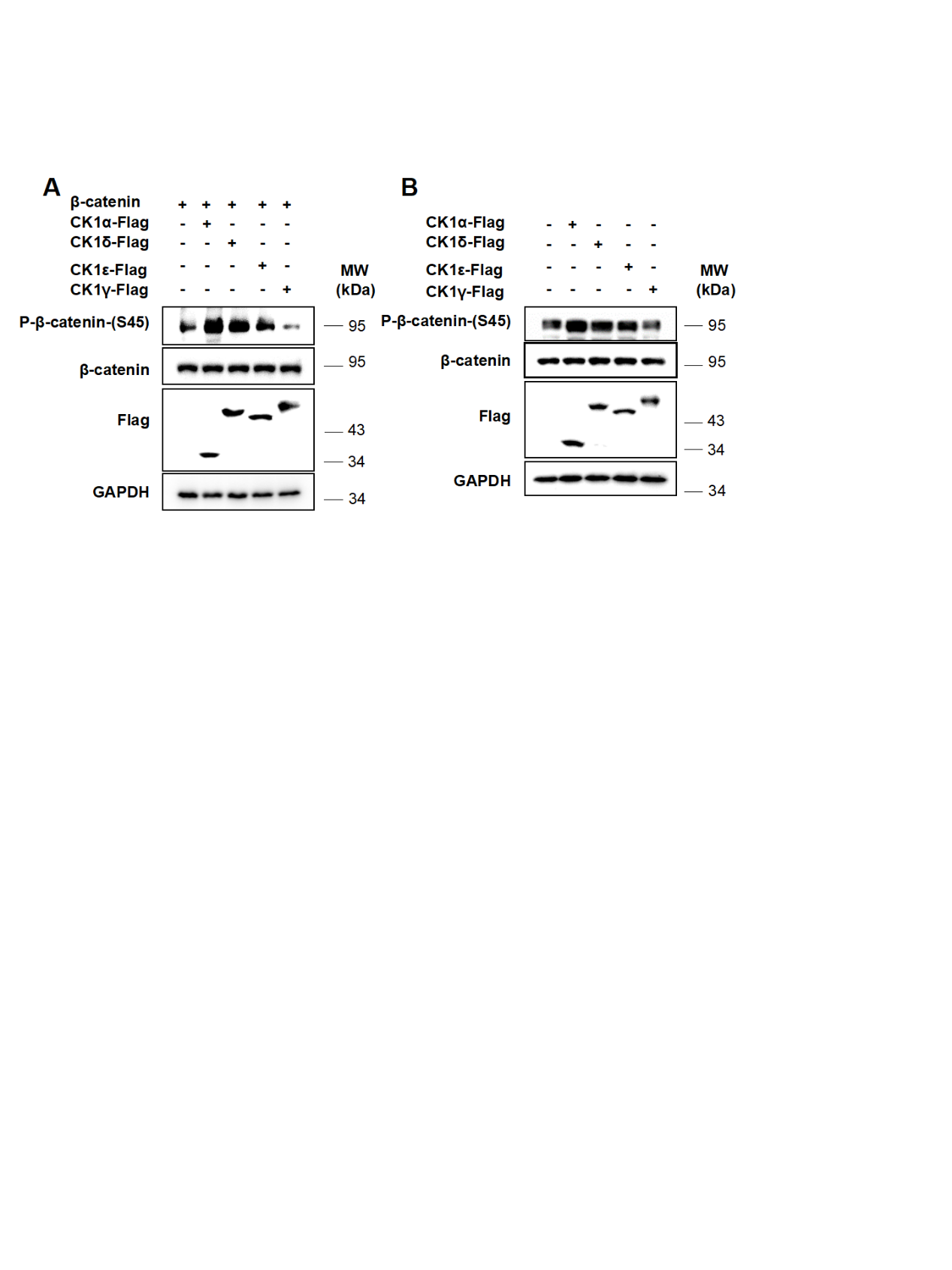


**Supplementary Figure 6.** The expression of CK1α or CK1δ or CK1ε induces the phosphorylation of β-catenin at S45. **(A)** HEK293T cells were transfected with β-catenin expression vector together with CK1α-Flag, CK1δ-Flag, CK1ε-Flag and CK1γ-Flag expression plasmids, respectively. **(B)** HEK293T cells were transfected with CK1α-Flag, CK1δ-Flag, CK1ε-Flag and CK1γ-Flag expression plasmids, respectively. Cell lysates were subjected to immunoblotting with the indicated antibodies.
